# Trophic assimilation efficiency markedly increases at higher trophic levels in four-level host–parasitoid food chain

**DOI:** 10.1098/rspb.2015.3043

**Published:** 2016-03-16

**Authors:** Dirk Sanders, Andrea Moser, Jason Newton, F. J. Frank van Veen

**Affiliations:** 1 Centre for Ecology and Conservation College of Life and Environmental Sciences, University of Exeter, Penryn Campus, Penryn, Cornwall TR10 9FE, UK; 2 Institute of Ecology and Evolution, University of Bern, Baltzerstrasse 6, Bern 3012, Switzerland; 3 NERC Life Sciences Mass Spectrometry Facility, Scottish Universities Environmental Research Centre, Rankine Avenue, Scottish Enterprise Technology Park, East Kilbride G75 0QF, UK

**Keywords:** *Alloxysta*, *Dendrocerus carpenteri*, *Coruna clavata*, biomass transfer, mummy parasitoids, stable isotope analysis

## Abstract

Trophic assimilation efficiency (conversion of resource biomass into consumer biomass) is thought to be a limiting factor for food chain length in natural communities. In host–parasitoid systems, which account for the majority of terrestrial consumer interactions, a high trophic assimilation efficiency may be expected at higher trophic levels because of the close match of resource composition of host tissue and the consumer's resource requirements, which would allow for longer food chains. We measured efficiency of biomass transfer along an aphid-primary–secondary–tertiary parasitoid food chain and used stable isotope analysis to confirm trophic levels. We show high efficiency in biomass transfer along the food chain. From the third to the fourth trophic level, the proportion of host biomass transferred was 45%, 65% and 73%, respectively, for three secondary parasitoid species. For two parasitoid species that can act at the fourth and fifth trophic levels, we show markedly increased trophic assimilation efficiencies at the higher trophic level, which increased from 45 to 63% and 73 to 93%, respectively. In common with other food chains, *δ*^15^N increased with trophic level, with trophic discrimination factors (Δ^15^N) 1.34 and 1.49‰ from primary parasitoids to endoparasitic and ectoparasitic secondary parasitoids, respectively, and 0.78‰ from secondary to tertiary parasitoids. Owing to the extraordinarily high efficiency of hyperparasitoids, cryptic higher trophic levels may exist in host–parasitoid communities, which could alter our understanding of the dynamics and drivers of community structure of these important systems.

## 1. Introduction

It has long been recognized [1] that food webs rarely have more than five trophic levels and most often fewer [2], constraining major aspects of food web structure [3]. A number of interacting factors, especially ecosystem size and primary productivity, are found to be related to food chain length [4–7]. An important mechanism behind these relationships is the (in)efficiency of transfer of productivity from one trophic level to the next, so that only large and/or highly productive ecosystems contain sufficient resources to sustain viable populations at high trophic levels [8–11]. A crucial component of this ecological efficiency is the trophic assimilation efficiency: the proportion of consumed resource biomass that is converted into consumer biomass. Theoretical work predicts trophic assimilation efficiency to be in the range of 13–50%, depending on predator–prey mass ratio [12], which is in accordance with the few empirical estimates that exist [13,14] and is generally assumed to be unrelated to trophic level or to decrease with increasing trophic level [15]. Trophic assimilation efficiency of consumer species can be an important factor determining ecosystem stability [16,17], as shown for lakes where during re-oligotrophication an increase in consumer assimilation efficiency resulted in a destabilizing increase in interaction strengths [18].

Host–parasitoid communities are widely used as model systems in population and multi-trophic community ecology as they arguably represent the majority of trophic interactions in terrestrial ecosystems [19], and a large body of ecological knowledge has been derived from these systems (e.g. [20–22]). Parasitoids are restricted to consuming a single host individual, representing a finite amount of resources with which to complete development from egg to adult, and there is therefore likely to be strong selection for using the resource with high efficiency [23]. Indeed, in host–parasitoid systems, trophic assimilation efficiency seems to be relatively high [23] and may be especially high for high trophic level hyperparasitoids (parasitoids whose hosts are also parasitoids) owing to the close match of resource content of the host and resource requirements of the hyperparasitoid, given their close phylogenetic relationships and similar lifestyles [24]. Therefore, we expect higher trophic assimilation efficiencies for species acting at higher trophic levels. It has been suggested that high competition among hyperparasitoids, and the fact that they are adapted to feeding on fellow Hymenoptera, may lead to frequent facultative tertiary and possibly even higher orders of parasitism [25,26]. However, there is a general assumption that, owing to physiological constraints, such interactions are negligibly rare in the wild. This assumption, and the fact that instances of higher-order parasitism are difficult to identify in the field, means that hyperparasitoids are generally treated as a fixed trophic level [27,28].

Here, we test this fundamental assumption by measuring assimilation efficiency along food chains in host–parasitoid systems. First, we used nitrogen stable isotope analysis (*δ*^15^N) [29] to test whether hyperparasitoids can truly act as tertiary parasitoids, feeding on other hyperparasitoids, as *δ*^15^N systematically increases with trophic level in other systems [30,31]. Then we measured efficiency of biomass transfer from primary parasitoid hosts to three hyperparasitoid species in the laboratory, and for two of these hyperparasitoid species we also measured the efficiency of biomass transfer when they feed on the other hyperparasitoid. We further compared carbon content and the carbon to nitrogen (C/N) ratio for the different trophic levels along the food chain, to test for the nutritional quality of the hosts at different trophic levels for the parasitoids. We use these data to test the hypothesis that trophic assimilation efficiency increases at the higher trophic level, reducing constraints on food chain length in host–parasitoid systems.

## 2. Material and methods

### (a) Study system

All species were collected in the field around Bern, Switzerland. Cultures were kept in climate chambers at 20/18°C with a 16 L : 8 D cycle. The primary parasitoid *Aphidius megourae* (Stary 1965) was reared on the aphid *Megoura viciae* (Buckton 1876) feeding on bean plants (*Vicia faba* L.). The larvae of primary aphid parasitoids first feed on the aphids' haemolymph and later kill the aphid by feeding on other tissues. They then pupate within the mummified skin of the aphid, creating the so-called mummy. They are commonly attacked by a diverse guild of hyperparasitoids belonging to two functional groups: (i) the secondary endophagous koinobiont parasitoids, which lay their eggs in the parasitoid larva within the still-living aphid, where they remain to hatch after mummification of the aphids [25] (from here on called endoparasitoids) and (ii) the so-called mummy parasitoids or secondary ectophagous idiobiont parasitoids, which attack their host at the pupal stage within the aphid mummy by depositing the eggs on the parasitoid host [26] (from here on mummy parasitoids). We used (i) the endoparasitoid *Alloxysta* sp. (Foerster 1869), and the two mummy parasitoids (ii) *Coruna clavata* (Walker 1833) and (iii) *Dendrocerus carpenteri* (Curtis 1829). *Alloxysta* lays an egg in the primary parasitoid larva in the still-living parasitized aphid host, where it remains and hatches only after mummification of the aphid [32]: this means the primary parasitoid larvae has stopped feeding on the aphid host, which allows us to estimate true trophic assimilation efficiencies even from the primary parasitoid to the next level (*Alloxysta*). All parasitoids used in this experiment had their host inside the aphid mummy as single resource as they were reared in individual gel capsules with no other resources available.

### (b) Study design and experimental set-up

**Figure 1.**
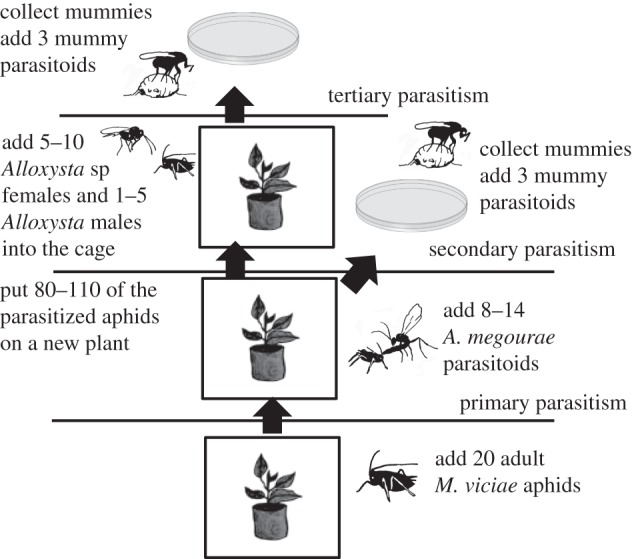
Experimental protocol for creating the food chains. Mummy parasitoids that were added to the food chain as secondary or tertiary parasitoids were either *D. carpenteri* or *C. clavata*.

One 14-day-old plant with 20 adult aphids was placed in each of 10 cages (24.5 × 24.5 × 24.5 cm, MegaView Science Co., Taiwan). Adult aphids were removed from cages after 3 days to obtain cohorts of 160–200 juveniles, which were parasitized by the parasitoid *A. megourae* (8–14 individuals added at day 6 and stayed for 48 h; parasitism rate 80–90%). Parasitized aphids were split at day 11: one-third were used to rear primary parasitoids and the other two-thirds were put onto another bean plant in a new cage with 5–10 female *Alloxysta* and 1–5 male *Alloxysta* (figure 1). At day 17 (for *A. megourae* cages) and day 20 (for *Alloxysta* cages), half of the mummies were transferred to Petri dishes together with three *D. carpenteri* or *C. clavata* females. After 48 h, the hyperparasitoids were removed from the Petri dishes to prevent multiple parasitism of hosts. Primary parasitoids started to eclose on day 20 after *A*. *megourae* attacked the aphids, secondary and tertiary parasitoids on days 31 and 36, respectively. We created the food chains in two separate runs: one with *D. carpenteri* and another with *C. clavata* as mummy parasitoid. Cages were daily checked for the formation of mummies, which were collected separately. After eclosure, individuals were stored in a freezer at −30°C.

Parasitoids were dried for 3 days at 65°C and then weighed (Sartorius Genius, ME ±0.01 mg). Individual weights of parasitoids were used to calculate the biomass transfer along the food chain and estimated as ‘individual biomass at higher trophic level/individual biomass at lower trophic level× 100’ (see statistical analysis for exact method). For stable isotope analysis, approximately 0.3–0.5 mg of dried insect material (two to eight individuals) was transferred into tin capsules (5 × 9 mm, HEKAtech GmbH, Germany). Then samples were combusted with an ECS 4010 elemental analyser (Costech, Milan, Italy) and analysed using a Delta V Plus isotope ratio mass spectrometer (Thermo Scientific, Bremen, Germany). For each sample, carbon content, nitrogen content and C/N ratio were measured alongside isotope ratios (*δ*^15^N and *δ*^13^C).

### (c) Statistical analyses

All statistical analyses were performed using R v. 3.1.0 [33]. Species- and trophic-level specific differences in dry weights and *δ*^15^N values were tested using linear models based on generalized least squares (errors are allowed to have unequal variances) provided by the nlme package [34]. We used VarIdent to account for variance heterogeneity in effect sizes between groups of parasitoids. For differences in hyperparasitoid weights according to the trophic levels and species, we specified the following six *a priori* contrasts [35], (i) the mummy parasitoid *C. clavata* secondary versus tertiary level, (ii) the mummy parasitoid *D. carpenteri* secondary versus tertiary level, (iii) the endoparasitoid versus mummy parasitoids as secondary parasitoids, (iv) the endoparasitoid versus mummy parasitoids as tertiary parasitoids, (v) *C. clavata* versus *D. carpenteri* as secondary parasitoids, and (vi) *C. clavata* versus *D. carpenteri* as tertiary parasitoids.

Biomass transfer efficiencies for all hyperparasitoid species were estimated from dry weight data. We used the function ‘sim’ from the R-package ‘arm’ [36] to simulate values from the posterior distribution of the species means, which were then used to estimate the proportions as derived parameters. A random sample of 5000 values from the posterior distribution of the model parameters (model: parasitoid dry weight ∼ parasitoid trophic group) was drawn for each trophic group (e.g. for *C. clavata* as secondary parasitoid). From these we estimated the 5000 values for the posterior distribution of the proportions ‘species A higher trophic level/ species B lower trophic levels’ for the pairs *D. carpenteri* and *C. clavata* acting at the different trophic levels versus their food base (*A. megourae* or *Alloxysta* sp.). We then tested for the posterior probability of the hypothesis that proportions (i) *D. carpenteri* tertiary/*Alloxysta* sp. > *D. carpenteri* secondary/*A. megourae* and (ii) *C. clavata* tertiary/*Alloxysta* sp. > *C. clavata* secondary/*A. megourae*. The Bayesian *p*-values presented in the results indicate the proportion of simulated values for which the hypothesis was true. Nitrogen content and C/N ratios in *A. megourae* versus *Alloxysta* as hosts for the mummy parasitoids were compared with the same Bayesian method.

For the stable isotope analysis, we tested for enrichment in ^15^N from (i) primary parasitoids versus secondary endoparasitoids and mummy parasitoids and (ii) the endoparasitoid *Alloxysta* sp. versus secondary mummy parasitoids.

The response variable, *δ*^15^N values for hyperparasitoids, was corrected against the base of the parasitoid food web (the mean for primary parasitoids for each experimental run), because primary parasitoid *δ*^15^N differed significantly by 1.1 ± 0.31‰ (*t*_1,14_ = −3.293, *p* = 0.0064) between the two experimental runs with *D. carpenteri* and *C. clavata*.

## 3. Results

### (a) Stable isotope analysis

**Figure 2.**
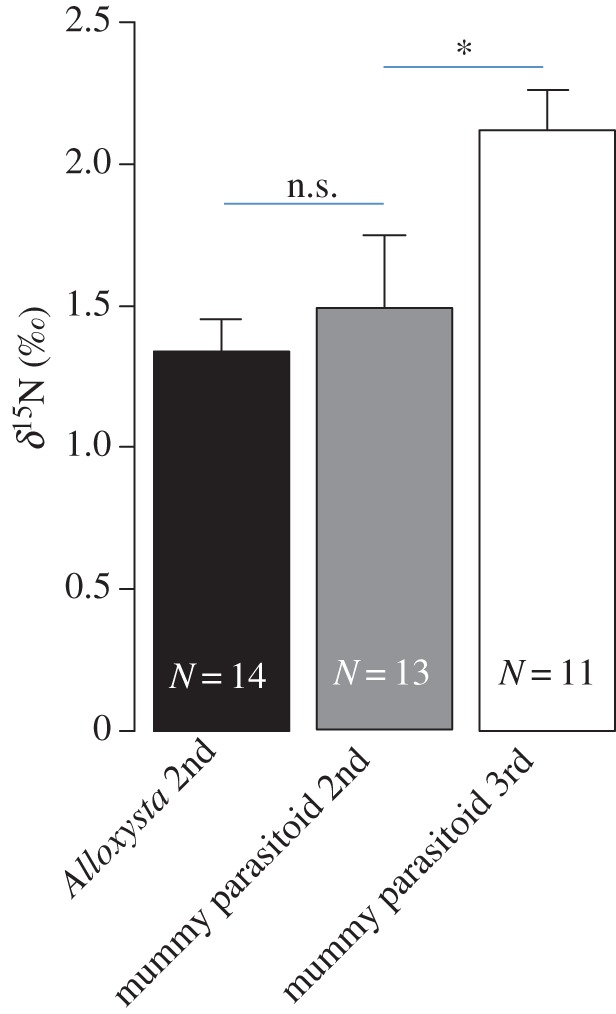
Mean *δ*^15^N values (+s.e.) of secondary and tertiary parasitoids displayed as enrichment to the mean value of the primary parasitoid *A. megourae* (including samples size). n.s., for a non-significance and *p < 0.05 for comparisons indicated by horizontal lines above the bars. (Online version in colour.)

We found a significant increase in ^15^N along the food chain with Δ^15^N = 1.34 (±0.11) and 1.49 (±0.25)‰ from primary parasitoids to endoparasitic and ectoparasitic secondary parasitoids, respectively, and 0.78 (±0.15)‰ from secondary to tertiary parasitoids (for details, see electronic supplementary material, Appendix S1). Both groups of secondary parasitoids, the endoparasitoid *Alloxysta* sp. and the mummy parasitoids, were similarly enriched in ^15^N (*t*_2,35_ = −0.54, *p*= 0.5864) but clearly separated from primary parasitoids (figure 2; *t*_1,35_= 5.86, *p* < 0.001). *δ*^15^N values significantly separated secondary mummy parasitoids from tertiary mummy parasitoids (figure 2, *t*_2,35_ = 2.15, *p* = 0.0381).

### (b) Biomass transfer between trophic levels

Body mass of *D. carpenteri* decreased significantly when acting as tertiary parasitoid compared with its mass when acting as secondary parasitoid (electronic supplementary material, Appendix S2; table 1). By contrast, we did not find a significant difference in mass between the two trophic levels of *C. clavata* (electronic supplementary material, Appendix S2; table 1). The mass of secondary *C. clavata* was significantly lower than the mass of secondary *D. carpenteri* with the same pattern when both were acting as tertiary parasitoids (electronic supplementary material, Appendix S2; table 1).

**Table 1.** Results for six *a priori* contrasts comparing the weights (in milligrams) of different parasitoid species and for *C. clavata* and *D. carpenteri* feeding at both the second and third level of parasitism.

species compared	value	s.e.	*t*-value	*p*-value
*C. clavata* 2nd to *C. clavata* 3rd	0.0058	0.0055	1.044	0.297
*D. carpenteri* 2nd to *D. carpenteri* 3rd	0.0124	0.0048	2.596	0.001
*Alloxysta* sp*.* to *C. clavata* 2nd and *D. carpenteri* 2nd	−0.0046	0.0040	−1.141	0.254
*Alloxysta* sp*.* to *C. clavata* 3rd and *D. carpenteri* 3rd	0.0135	0.0037	3.610	<0.001
*C. clavata* 2nd to *D. carpenteri* 2nd	−0.0309	0.0056	−5.474	<0.0001
*C. clavata* 3rd to *D. carpenteri* 3rd	−0.0243	0.0046	−5.276	<0.0001

**Figure 3.**
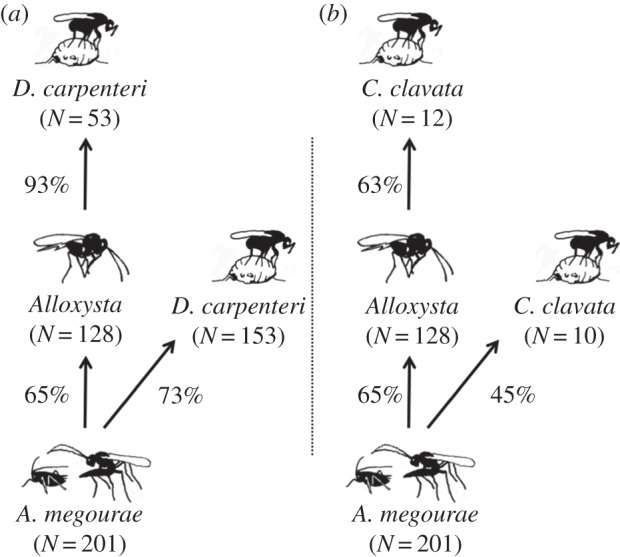
Biomass transfer (% of dry weight) from one trophic level to the next higher level along the primary parasitoid–secondary parasitoid–tertiary parasitoid trophic chain for the mummy parasitoids (*a*) *D. carpenteri* and (*b*) *C. clavata*. The sample size is given in brackets.

*D. carpenteri* (acting as secondary or tertiary parasitoid) was more efficient than *Alloxysta* or *C. clavata* (figure 3, table 1). *D. carpenteri* converted 73% of the host's body mass as secondary parasitoids and remarkably, 93% of the host's body mass when acting as tertiary parasitoid (figure 3, posterior probability of 0.999 that the efficiency is higher for *D. carpenteri* at the tertiary level). *C. clavata* also showed higher efficiency when acting as tertiary parasitoid, with 45% as secondary and 63% as tertiary (figure 3, posterior probability of 0.999 for higher efficiency at tertiary level) but with less efficiency than *D. carpenteri* (figure 3, posterior probability of 1 for higher efficiency in *D. carpenteri* for both trophic levels). The nitrogen content was 1.19 times higher in *Alloxysta* than in *A. megourae* (electronic supplementary material, Appendix S3, posterior probability of 1 for higher content in *Alloxysta*) and the C/N ratio dropped from 5.28 in *A. megourae* to 4.21 in *Alloxysta* (posterior probability of 0.999 for lower ratio in *Alloxysta*).

## 4. Discussion

We found extraordinarily high trophic assimilation efficiency for hyperparasitoids, which markedly increased along the trophic chain for both mummy parasitoids *D. carpenteri* and *C. clavata*. Both species can act at the secondary and tertiary parasitism level but were far more efficient at the tertiary level in converting host biomass. Differences in ^15^N enrichment allowed us to confirm that both species of mummy parasitoids were capable of acting as true tertiary parasitoids. The high efficiency of biomass transfer indicates there is no physiological barrier to these intraguild interactions between hyperparasitoids at higher trophic levels, thereby falsifying the assumption that there are strong constraints on food chain length in host–parasitoid food webs.

These results have significant implications for our understanding of these important systems. In addition to predicted effects of assimilation efficiency on community stability [16–18], constraints on food chain length have been shown to explain many of the universal properties found in network structure among food webs [3]. The possibility of cryptic higher trophic levels, owing to relaxation of these constraints, therefore, also means that host–parasitoid networks may contain a hidden structure that is fundamentally different from other food webs, with implications for community dynamics and stability [27].

Our results suggest that the higher up in the trophic chain a hyperparasitoid acts, the more easily it can convert the host tissue. A possible reason for this is that unprofitable food components have already been removed earlier from the food source and plant allelochemicals diluted, benefiting insect predators and parasitoids [37,38]. Plant defensive chemicals may be assimilated at the first parasitism level [39], but not passed on to the higher trophic levels. Towards the top end of the food chain, nitrogen tends to be concentrated leading to a closer match between the nutritional content of host and the nutritional requirements of consumer [23]. And indeed, the nitrogen content was higher with the C/N ratio consequently being lower in the body of *Alloxysta*, the host for the tertiary parasitoids, than in *A. megourae*, the host for the secondary parasitoids. Interestingly, the C/N ratios of the mummy parasitoids were very similar to that of *Alloxysta* (electronic supplementary material, Appendix S3). For the mummy parasitoids *Alloxysta* as host can be more efficiently exploited than the primary parasitoid, leading to the higher trophic assimilation efficiency at the higher trophic level. Therefore, *D. carpenteri* and *C. clavata* were far more efficient as tertiary parasitoids than as secondary parasitoids.

It has been suggested that higher trophic levels in arthropod communities contain progressively fewer lipids and more protein in their bodies, which makes carbohydrate and fat less available for higher-order consumers and potentially limiting the number of trophic levels [40,41]. However, it appears that in host–parasitoid systems the efficiency of host exploitation is high and fatty acids are consumed directly from the host without modification, leading to stable fatty acid compositions throughout the food chains [42]. C/N ratios were very similar for all hyperparasitoids in our study, suggesting stable carbon availability even at higher trophic levels.

*D. carpenteri* was more efficient in converting host biomass than *C. clavata*. *C. clavata* shows host-feeding prior to oviposition to accumulate enough protein to produce eggs owing to lack of energy uptake as a larva [43]. Therefore, selection pressure for high efficiency should be more pronounced for *D. carpenteri*. Parasitoids are further capable of taking up sugar in the wild from sources such as honeydew, nectar and extra floral nectar [44].

Owing to the extraordinary efficiency of parasitoids at high trophic levels, cryptic higher trophic levels may exist in host–parasitoid communities, which could alter our understanding of the dynamics and drivers of community structure of these important systems. Stable isotope analysis can be used to study the vertical trophic structure of parasitoids in the field in order to reveal this hidden aspect of the food webs.

## Supplementary Material

Mean δ15N and δ13C values; Mean dry weight of parasitoids

## Supplementary Material

Mean percentage of Carbon and Nitrogen and the C/N ratio

## Data accessibility

The datasets supporting this article have been uploaded as part of the electronic supplementary material, Appendix S4.
