# Supplementary material for: Trophic assimilation efficiency markedly increases at higher trophic levels in four-level host–parasitoid food chain
Source: Proc Biol Sci. 2016 Mar 16;283(1826):20153043. doi: 10.1098/rspb.2015.3043 (PMC4810866; doi:10.1098/rspb.2015.3043)
Supplement: Mean δ15N and δ13C values; Mean dry weight of parasitoids [file rspb20153043supp1.docx]

**Supplementary material**

**Appendix 1** Mean δ^15^N and δ^13^C values (±SE) and sample number (n) for each of the trophic groups.

| Experiment | Group | δ^15^N ‰ | δ^13^C ‰ | n |
| --- | --- | --- | --- | --- |
| 1 | Plants | 2.74 ±0.11 | -30.53 ±0.30 | 5 |
|  | Aphids | 2.56 ±0.16 | -28.30 ±0.28 | 4 |
|  | Primary parasitoids | 4.22 ±0.10 | -29.99 ±0.23 | 5 |
|  | *Alloxysta* | 5.47 ±0.15 | -28.30 ±0.31 | 5 |
|  | *C. clavata* secondary | 5.64 ±0.59 | -29.46 ±0.58 | 3 |
|  | *C. clavata* tertiary | 6.19 ±0.21 | -29.08 ±0.27 | 5 |
| 2 | Primary parasitoids | 3.20 ±0.29 | -30.77 ±0.43 | 9 |
|  | *Alloxysta* | 4.59 ±0.15 | -31.04 ±0.31 | 9 |
|  | *D. carpenteri* secondary | 4.72 ±0.30 | -30.59 ±0.43 | 10 |
|  | *D. carpenteri* tertiary | 5.45 ±0.20 | -30.13 ±0.43 | 6 |

**Appendix 2** Mean dry weight (mg) (±SE) of primary parasitoids, endoparasitoids and both mummy parasitoid species as secondary and tertiary parasitoids (for statistical tests see Table 1).


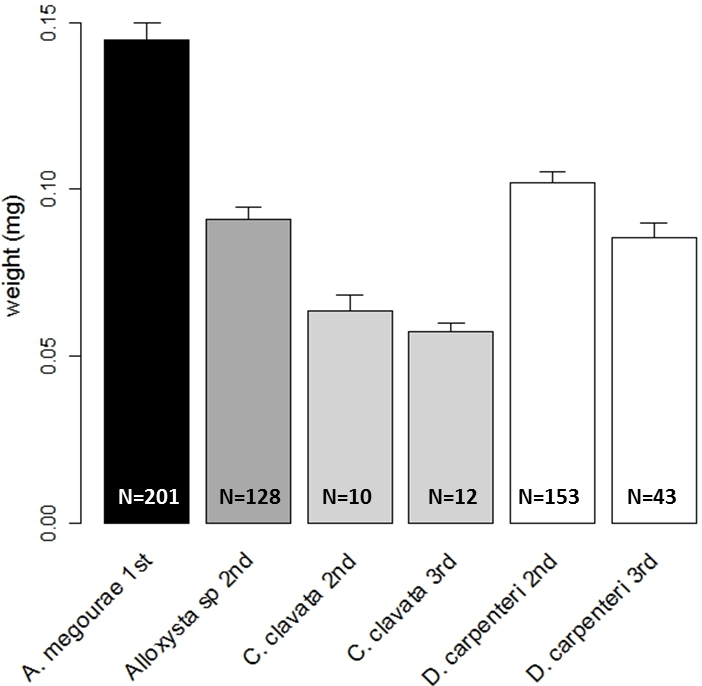


**Appendix 3** Mean and standard error (SE) for percentage of Carbon and Nitrogen and the C/N ratio in samples from the different trophic groups (aphids, primary, secondary and tertiary parasitoids).

|  | Percentage of C | | Percentage of N | | C/N ratio | |
| --- | --- | --- | --- | --- | --- | --- |
|  | Mean | SE | Mean | SE | Mean | SE |
| Aphid (M. viciae) | 51.19 | 1.74 | 7.37 | 0.24 | 6.97 | 0.28 |
| A. megourae (primary) | 55.44 | 0.63 | 10.57 | 0.19 | 5.28 | 0.13 |
| Alloxysta (secondary) | 52.20 | 1.00 | 12.59 | 0.28 | 4.21 | 0.17 |
| D. carpenteri (secondary) | 53.44 | 0.93 | 11.26 | 0.21 | 4.78 | 0.14 |
| D. carpenteri (tertiary) | 52.04 | 0.74 | 11.20 | 0.35 | 4.66 | 0.09 |
| C. clavata (secondary) | 54.61 | 5.32 | 13.08 | 0.25 | 4.17 | 0.36 |
| C. clavata 3^rd^ (tertiary) | 50.28 | 2.74 | 11.89 | 0.44 | 4.25 | 0.28 |
